# Selenium Attenuates TBHP-Induced Apoptosis of Nucleus Pulposus Cells by Suppressing Mitochondrial Fission through Activating Nuclear Factor Erythroid 2-Related Factor 2

**DOI:** 10.1155/2022/7531788

**Published:** 2022-04-11

**Authors:** Peng Wang, Shuo Zhang, Weijian Liu, Songfeng Chen, Xiao Lv, Binwu Hu, Zengwu Shao

**Affiliations:** ^1^Department of Orthopaedics, Union Hospital, Tongji Medical College, Huazhong University of Science and Technology, Wuhan 430022, China; ^2^Department of Orthopaedics, The First Affiliated Hospital of Zhengzhou University, Zhengzhou 450052, China

## Abstract

Intervertebral disc (IVD) degeneration (IDD), the leading cause of low back pain (LBP), remains intractable due to a lack of effective therapeutic strategies. Several lines of studies have documented that nucleus pulposus cell (NPC) death induced by excessive oxidative stress is a crucial contributor to IDD. However, the concrete role and regulation mechanisms have not been fully clarified. Selenium (Se), a vital prosthetic group of antioxidant enzymes, is indispensable for maintaining redox homeostasis and promoting cell survival. However, no light was shed on the role of Se on IDD progression, especially regulation on mitochondrial dynamics and homeostasis. To fill this research gap, the current study focuses on the effects of Se, including sodium selenite (SS) and selenomethionine (Se-Met), on IDD progression and the underlying mechanisms. In vitro, we found that both SS and Se-Met alleviated tert-butyl hydroperoxide- (TBHP-) induced oxidative stress, protected mitochondrial function, and inhibited apoptosis of NPCs. Further experiments indicated that Se suppressed TBHP-induced mitochondrial fission and rescued the imbalance of mitochondrial dynamics. Promoting mitochondrial fission by carbonyl cyanide 4-(trifluoromethoxy) phenylhydrazone (FCCP) partially counteracted the cytoprotective effects of Se. Moreover, blocking nuclear factor erythroid 2-related factor 2 (Nrf2) with ML385 proved that the effect of Se on regulating mitochondrial dynamics was attributed to the activation of the Nrf2 pathway. In the puncture-induced rat IDD model, a supplement of Se-Met ameliorated degenerative manifestations. Taken together, our results demonstrated that Se suppressed TBHP-induced oxidative stress and mitochondrial fission by activating the Nrf2 pathway, thereby inhibiting the apoptosis of NPCs and ameliorating IDD. Regulation of mitochondrial dynamics by Se may have a potential application value in attenuating the pathological process of IDD.

## 1. Introduction

Low back pain (LBP), the leading cause of disability worldwide, imposes an enormous economic burden on both healthcare and social support systems [1, 2]. Intervertebral disc (IVD) degeneration (IDD) has been reported to be the primary contributor to LBP [3, 4]. Nucleus pulposus (NP), mainly constituted by NP cells (NPCs) and extracellular matrix (ECM), plays a pivotal role in maintaining the integrity and biomechanical equilibrium of IVD [5, 6]. Previous studies have demonstrated that the decline of NPC quantity and quality is one of the major contributors to IDD [7–9]. Excessive death of NPCs resulted in the reduction of ECM synthesis with diminished water-binding ability, eventually triggering IDD. Nevertheless, the precise underlying mechanisms of IDD have not yet been fully elucidated, and effective interventions are still lacking.

Recently, ample compelling evidence indicated that oxidative stress is closely associated with the occurrence and development of IDD [10–12]. When the dynamic balance between reactive oxygen species (ROS) generation and antioxidant defense is disrupted in some pathological conditions, oxidative stress may occur and then lead to IDD [13–15]. Excessive oxidative stress triggers mitochondrial dysfunction, mitochondrial membrane potential collapse, and ATP production decline, finally contributing to apoptosis and senescence of NPCs [15–17]. Therefore, alleviating oxidative stress and maintaining mitochondrial homeostasis in NPCs are supposed to be promising therapeutic strategies for IDD.

Mitochondria are highly dynamic cellular organelles that undergo continuous fusion and fission, a process termed mitochondrial dynamics [18]. When cells suffer metabolic or environmental stress, mitochondrial fusion is activated to promote the complementation of damaged mitochondria, and fission contributes to quality control by removing the damaged mitochondria. However, inordinate cellular stress triggers excessive mitochondrial fission, which results in cell apoptosis [19]. Suppressing a high level of mitochondrial fission can rescue defective mitochondria and dramatically increase antioxidative defense [19]. Disrupted mitochondrial dynamics were proved to contribute to apoptosis and senescence of NPCs, while remodeling mitochondrial dynamics was beneficial to NPC survival [20]. Therefore, it is a novel and crucial insight to further explore IDD protection strategies based on regulating mitochondrial dynamics.

Nuclear factor erythroid 2-related factor 2 (Nrf2), a main regulator of the endogenous defense system, confers adaptive protection against oxidative stress via regulating the expression of a series of cytoprotective genes [21, 22]. Under stressed conditions, Nrf2 transfers into the nucleus after being released from Kelch-like ECH-associated protein 1 (Keap1) and then facilitates the expression of heme oxygenase-1 (HO-1) and superoxide dismutase 2 (SOD2), which protect cells from oxidative injury [23, 24]. In addition, Nrf2 can maintain mitochondrial homeostasis and function when oxidative stress occurs [25–27]. Recently, therapies targeting Nrf2 were also reported to play essential roles in alleviating oxidative stress-induced IDD [10, 21, 28].

Selenium (Se) serves as an indispensable component of antioxidative enzymes, such as glutathione peroxidase (GPX) and thioredoxin reductase (TRXR) [29]. Se was reported to confer various protective effects in the body, such as anti-inflammatory, antioxidative, and antiapoptotic effects [30–33]. Zhang et al. revealed that Se shielded mouse kidneys from zearalenone-induced oxidative stress and apoptosis [34]. Moreover, Se has been proved to protect chicken hepatocytes against cadmium-induced autophagy and apoptosis through Nrf2-mediated protection [35]. Considering that oxidative stress and apoptosis of NPCs are major contributors to IDD, it is hypothesized that Se may serve as a promising therapeutic agent in alleviating IDD. To our best knowledge, studies regarding the association of Se and IDD are scarce.

To fill this research gap, the current study explored the antioxidative and antiapoptotic effects of Se against TBHP-induced damages in NPCs and whether the Nrf2 signaling pathway could be activated by Se to attenuate mitochondrial fission. Furthermore, we also evaluated whether Se-Met could attenuate puncture-induced IDD in vivo. Taken together, our study further discussed the mechanisms of regulation of oxidative stress and mitochondrial dynamics in NPCs and highlighted the therapeutic potential of Se for relieving IDD.

## 2. Materials and Methods

### 2.1. Rat Primary Nucleus Pulposus Cell Isolation and Culture

Sprague-Dawley rats (weighing 200-250 g) were purchased from the Experimental Animal Center of Tongji Medical College, Huazhong University of Science and Technology (Wuhan, China). Primary NPCs were isolated from the tails of rats after being euthanized by intraperitoneal injection of an overdose of pentobarbital as described previously [36]. Briefly, NP tissues were mechanically cut into pieces and digested with 0.2% (*m*/*v*) type II collagenase (Sigma-Aldrich, St. Louis, MO, USA) at 37°C for 15 min. After centrifugation and resuspension, the NPCs were seeded into culture flasks in DMEM/F-12 (HyClone, Logan, UT, USA) supplemented with 10% fetal bovine serum (FBS; Gibco, Grand Island, NY, USA) and 1% penicillin/streptomycin and then cultured at 37°C in an incubator. The culture medium was changed every three days. NPCs were harvested by trypsinization after achieving 80-90% confluence for subculture. The second passage of cells was used for the following experiments.

### 2.2. Treatment of NPCs

NPCs were planted into culture plates at appropriate densities and cultured with a complete medium for 72 h. To evaluate the cytotoxicity of Se on NPCs, NPCs were treated with different concentrations of SS (0.5, 1, 3, 5, 7.5, 15, and 30 *μ*M) (Sigma-Aldrich) or Se-Met (25, 50, 75, 100, 150, 300, and 500 *μ*M) (MCE, NJ, USA) for 24 h. In addition, NPCs were treated with different concentrations of TBHP (25, 50, 75, 100, 125, and 150 *μ*M) for 6 h to select the appropriate working concentration. To assess the effect of Se on the NPC viability under oxidative stress, NPCs were pretreated with different concentrations of SS or Se-Met for 24 h and then incubated with TBHP for another 6 h. Carbonyl cyanide 4-(trifluoromethoxy) phenylhydrazone (FCCP) (Selleck Chemicals, Houston, TX, USA) is an uncoupler of mitochondrial oxidative phosphorylation and an agonist of mitochondrial fission. To further explore the role of mitochondrial dynamics in TBHP-induced cell damages, NPCs were administrated with Se solely or in combination with 5 *μ*M FCCP for 24 h and then treated with TBHP (100 *μ*M) for another 6 h. Nrf2 inhibitor ML385 (10 *μ*M) (Selleck Chemicals) was used to evaluate the role of the Nrf2 pathway in NPCs.

### 2.3. Cell Viability Assay

Cell viability was measured using Cell Counting Kit-8 (CCK-8) assays. NPCs were planted into 96-well plates at the appropriate density. After treatment, the culture medium was changed to a 100 *μ*L DMEM/F-12 serum-free medium with 10 *μ*L CCK-8 solution and incubated for 2 h at 37°C. Cell viability was quantified by measuring the absorbance at 450 nm using a microplate reader (Thermo Scientific, MA, USA).

### 2.4. Western Blot Assay

The total protein of NPCs was extracted by lysing in the radioimmunoprecipitation assay (RIPA) lysis buffer (Beyotime, Shanghai, China) supplemented with a cocktail of proteases and phosphatase inhibitors. Protein concentration was determined with the Enhanced BCA Protein Assay Kit (Beyotime). Subsequently, the protein was electrophoretically separated via sodium dodecyl sulfate-polyacrylamide gel (SDS-PAGE) and transferred onto polyvinylidene difluoride (PVDF) membranes (EMD Millipore, Billerica, MA, USA). After being blocked with 5% skimmed milk for 1 h at room temperature, the PVDF membranes were incubated overnight at 4°C with the primary antibodies against Nrf2 (1 : 1000, ProteinTech, Wuhan, China), HO-1 (1 : 1000, ProteinTech), SOD2 (1 : 2000, ProteinTech), dynamin-related protein 1 (DRP1) (1 : 1000, Affinity Biosciences, OH, USA), mitochondrial fission factor (MFF) (1 : 1000, ProteinTech), mitochondrial fission 1 (Fis1) (1 : 1000, ProteinTech), optic atrophy 1 (OPA1) (1 : 1000, ProteinTech), mitofusin 1 (Mfn1) (1 : 1000, ProteinTech), mitofusin 2 (Mfn2) (1 : 1000, ProteinTech), Aggrecan (1 : 500, NOVUS, USA), collagen II (1 : 1000, Affinity Biosciences), matrix metalloproteinase 3 (MMP3) (1 : 1000, Affinity Biosciences), MMP9 (1 : 1000, Affinity Biosciences), cleaved-caspase 3 (1 : 1000, Affinity Biosciences), caspase 9 (1 : 1000, ProteinTech), Bcl-2 (1 : 1000, Abcam, Cambridge, MA, USA), Bax (1 : 1000, ProteinTech), and *β*-actin (1 : 2000, Cell Signaling Technology, Danvers, MA, USA). Then, the PVDF membranes were washed three times with Tris-buffered saline containing Tween 20 (TBST) buffer for 5 minutes per time and incubated with the respective secondary antibodies for 1 h at room temperature. Finally, the protein bands were visualized with an electrochemiluminescence (ECL) detection reagent (Affinity Biosciences) and analyzed by the ImageJ software (Bethesda, MD, USA).

### 2.5. Live/Dead Cell Staining

Calcein acetoxymethyl ester (Calcein-AM) (Santa Cruz Biotechnology, Inc., Dallas, TX, USA) and propidium iodide (PI) (Nanjing Keygen Biotech, Nanjing, China) were used to detect live and dead NPCs, respectively. After treatment, primary NPCs cultured in 12-well plates were washed once with phosphate-buffered saline (PBS) and incubated for 30 minutes with Calcein-AM (2 *μ*M) at 37°C. Subsequently, cells were gently rinsed with PBS and then incubated with PI (1 : 1000, dissolved in serum-free medium) for 10 minutes at 37°C in the dark. After staining, the NPCs were washed with PBS and photographed by fluorescence microscopy (Olympus IX71, Japan). Under fluorescence microscopy, live cells fluoresced green, and dead cells fluoresced red.

### 2.6. Terminal Deoxynucleotidyl Transferase Biotin-dUTP Nick End Labeling (TUNEL) Assay

Apoptosis of NPCs was detected by TUNEL assays using the One-Step TUNEL Apoptosis Assay Kit (Beyotime). NPCs were seeded on glass coverslips in 12-well plates and then conducted with corresponding treatments. After being fixed with 4% formaldehyde for 30 minutes and permeabilized with 0.3% Triton X-100 (Beyotime) for 15 minutes, NPCs were incubated with TUNEL inspection fluid for 1 h in the dark. Then, NPCs were washed once with PBS followed by incubation with 4′,6-diamidino-2-phenylindole (DAPI) for 10 minutes in the dark. Finally, the slides were observed under a fluorescence microscope (Olympus BX53, Japan). NPCs with red fluorescence were considered apoptotic cells.

### 2.7. Immunofluorescence (IF) Staining

After treatments, slides containing NPCs were fixed with 4% formaldehyde for 30 minutes and permeabilized with 0.3% Triton X-100 for 15 minutes at room temperature. After blocking with goat serum albumin for 1 h, samples were rinsed with PBS three times and incubated overnight at 4°C with primary antibodies against Nrf2 (1 : 300, ProteinTech) and TOM-20 (1 : 200, Cell Signaling Technology). Then, cells were incubated with fluorophore-conjugated secondary antibody (1 : 200, Servicebio, Wuhan, China) for 1 h at room temperature in the dark. Subsequently, the nuclei were stained with DAPI for 10 minutes. Images were captured by a fluorescence microscope (Olympus BX53).

### 2.8. Flow Cytometry Analysis

According to the manufacturer's instructions, the Annexin V-FITC/PI Apoptosis Detection kit (Beyotime), DCFH-DA (Beyotime), MitoSOX Red (Thermo Fisher Scientific, Waltham, MA, USA), and JC-1 Assay Kit (Beyotime) were applied to detect apoptosis levels, the production of cellular ROS and mitochondrial ROS, and mitochondrial membrane potential levels in NPCs, respectively. In brief, NPCs were harvested by trypsinization after treatment and then centrifuged and washed three times with PBS. Then, NPCs were labeled with the above-mentioned kits and detected by a flow cytometer, and the results were analyzed by the FlowJo software (Tree Star, Inc. San Carlos, CA, USA).

### 2.9. ATP Production Assay

An Enhanced ATP Assay Kit (Beyotime) was used to measure the ATP content of NPCs after different treatments. NPCs were lysed with the ATP lysis buffer and then centrifuged at 12,000 g for 5 minutes. After that, the supernatants were collected and reacted with the ATP detection working dilution. Meanwhile, cellular protein concentration was quantified by an Enhanced BCA Protein Assay Kit (Beyotime) as previously stated. Finally, luminescence spectrometry (PerkinElmer) was used to detect luminescence activity. The ATP level of NPCs was normalized to cellular protein concentration.

### 2.10. Malondialdehyde (MDA) Detection

The MDA concentration of NPCs was detected by the Lipid Peroxidation MDA Assay Kit (Beyotime). Briefly, after treatment, NPCs were lysed with lysis buffer and then centrifuged at 12,000 g for 10 minutes. Subsequently, the supernatants were collected and reacted with the MDA detection working dilution for 15 minutes at 100°C. After cooling to room temperature, the mixture of supernatants was centrifuged at 1000 g for 10 minutes. Finally, the supernatants were added to the 96-well plate to measure the absorbance at 532 nm using a spectrophotometer (PerkinElmer). The MDA level of NPCs was normalized to cellular protein concentration.

### 2.11. Puncture-Induced Rat IDD Model

All animal operations were performed in strict compliance with the medical ethics committee of Tongji Medical College, Huazhong University of Science and Technology. 21 male Sprague-Dawley rats (weighing 200-250 g) were obtained from the Experimental Animal Center of Tongji Medical College, Huazhong University of Science and Technology (Wuhan, China). With the help of the digital table method, rats were randomly distributed into three groups: sham group (*n* = 7), IDD group (*n* = 7), and Se-Met group (*n* = 7). A previously reported puncture-induced rat IDD model was employed in the current study [37, 38]. Rats in all three groups were anesthetized by intraperitoneal injection of pentobarbital (50 mg/kg), and then coccygeal 6/7 IVD of each rat tail in the IDD group and the Se-Met group were located by trial radiography. Subsequently, 27-gauge needles were used to perforate the whole layer of the AF, in parallel to the endplates. The needle was then rotated 360° and held in the disc for 1 minute. After puncturing, the rats in the Se-Met group were treated with Se-Met (2 mg/kg/day) by intragastric administration, and rats in the sham group and the IDD group were treated with an equal volume of deionized water every day for 4 weeks. After rats were euthanatized with an overdose of phenobarbital, the corresponding discs were harvested and fixed in 4% paraformaldehyde for 48 h, followed by decalcification with 10% ethylenediaminetetraacetic acid solution and embedded in paraffin.

### 2.12. Histological Analysis

The paraffin-embedded discs were cut into 4 *μ*m-thick sections. For histology, the sections were deparaffinized, rehydrated, and then stained with hematoxylin and eosin (HE) and Safranin O-Fast Green (SO). In addition, the histopathological grading scores were assessed according to the grading scale [39]. For IHC staining, the sections were deparaffinized, rehydrated, and incubated with 3% H_2_O_2_ for 20 minutes at room temperature to eliminate endogenous peroxidase activity. Then, the antigen was retrieved by pressure cooking in 10 mmol/L citrate buffer, pH = 6. Subsequently, the sections were blocked with 10% goat serum albumin for 30 min at room temperature. The sections were then incubated with a primary antibody against Nrf2 (1 : 400, ProteinTech), collagen II (1 : 50, Affinity Biosciences), and MMP13 (1 : 300, ProteinTech) at 4°C overnight. Sections incubated with nonspecific IgG were used as negative controls. Next, the sections were incubated with horseradish peroxidase-conjugated goat anti-rabbit secondary antibody for 1 h at 37°C and counterstained with hematoxylin. For the gray-scale statistics of IHC staining, we used ImageJ to calculate the integrated optical density (IOD) of IHC images.

### 2.13. Statistical Analysis

In the current study, all experiments were repeated at least three times independently. Data were analyzed using the GraphPad Prism V.8.0 software (GraphPad Software, San Diego, CA, USA), and the results were presented as mean ± standard deviation (SD). The student's *t*-test was used for comparisons between two independent groups, while for comparisons of more than two groups, one-way analysis of variance (ANOVA) was used and followed by Tukey's post hoc test. *P* values < 0.05 were taken to be statistically significant.

## 3. Results

### 3.1. Se Protected NPCs from TBHP-Induced Cytotoxicity

**Figure 1:**
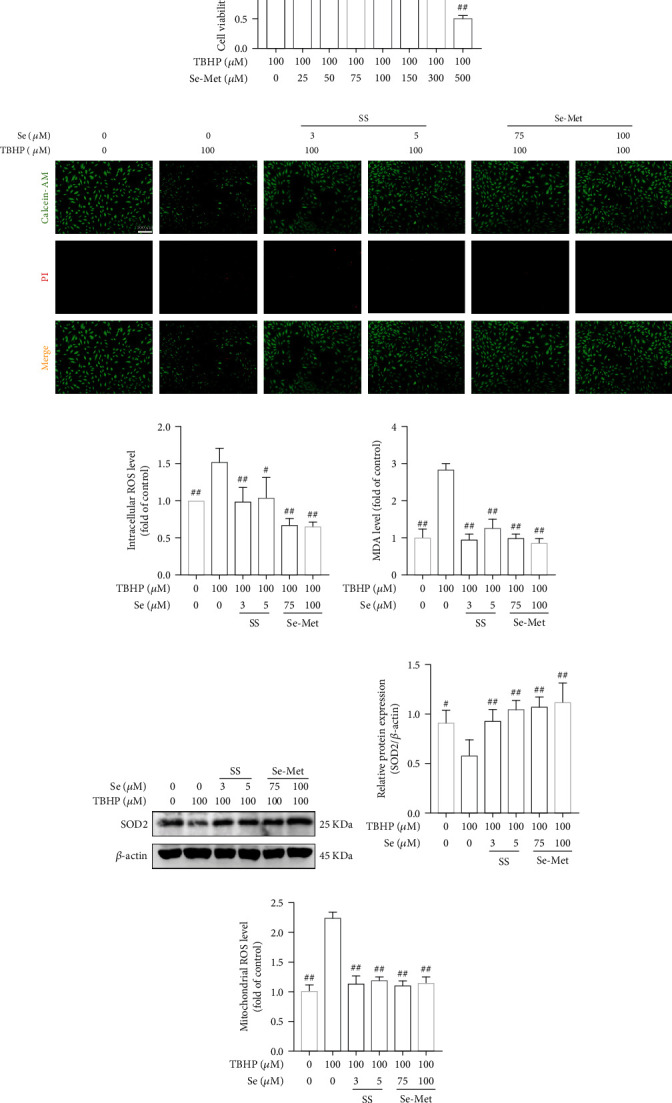
Se alleviated TBHP-induced cytotoxicity and restored mitochondrial function in NPCs. (a, b) CCK-8 result of the NPCs viability with different concentrations of SS or Se-Met for 24 h. (c) CCK-8 result of the NPCs viability with different concentrations of TBHP for 6 h. (d, e) CCK-8 result of NPCs with TBHP (100 *μ*M) and different concentrations of SS and Se-Met, respectively. (f) Fluorescence photomicrograph of live/dead cell staining of NPCs treated with Se and TBHP (scale bar: 100 *μ*m). Green fluorescent indicates live cells and red fluorescent indicates dead cells. (g) Intracellular ROS measured by flow cytometry analysis. (h) Intracellular MDA levels in NPCs. (i, j) The expression of SOD2 in NPCs detected by western blot. (k) The mitochondrial ROS levels measured by flow cytometry analysis. (l) Mitochondrial membrane potential levels measured by flow cytometry analysis. (m) Quantitation of the ratio of JC-1 monomer. (n) Intracellular ATP content in NPCs. The data are represented as the mean ± SD from at least 3 independent experiments. ^∗^*P* < 0.05 and ^∗∗^*P* < 0.01 vs. control, ^#^*P* < 0.05 and ^##^*P* < 0.01 vs. the TBHP group. NS: no statistically significant difference.

As shown in Figures 1(a) and 1(b), SS and Se-Met presented cytotoxic effects at concentrations greater than 15 *μ*M and 300 *μ*M, respectively. Under TBHP treatment, the cell viability of NPCs was significantly decreased in a dose-dependent manner (Figure 1(c)). Then 100 *μ*M TBHP was selected for subsequent experiments.

The counteracting effects of both SS and Se-Met on TBHP (100 *μ*M) cytotoxicity were evaluated in NPCs. As shown in Figure 1(d) 3 *μ*M and 5 *μ*M SS presented remarkable protective effect, while 75 *μ*M and 100 *μ*M Se-Met obviously increased the cell viability of NPCs (Figure 1(e)). Therefore, the above-mentioned concentrations of Se were applied in the following experiments. In addition, live/dead cell staining showed that TBHP treatment decreased the ratio of alive cells (green fluorescence) and increased the ratio of dead cells (red fluorescence), whereas Se reversed the changes (Figure 1(f)).

### 3.2. Se Inhibited TBHP-Induced Oxidative Stress and Restored Mitochondrial Function

Flow cytometry analysis indicated that the production of intracellular ROS in the TBHP group was significantly higher than the control group (*P* < 0.01), and Se greatly attenuated the elevation induced by TBHP (Figure 1(g) and Figure S1(a)). Consistently, Se markedly suppressed the TBHP-induced increase of MDA, an indicator of lipid peroxidation (Figure 1(h)). In addition, Se enhanced the expression of the antioxidant enzyme SOD2 in NPCs exposed to TBHP (Figures 1(i) and 1(j)).

Excessive oxidative stress has been widely known as the main cause of mitochondrial damage, which contributes to mitochondrial dysfunction. As shown in Figure 1(k) and Figure S1(b), Se reduced the excessive production of mitochondrial ROS caused by TBHP. Furthermore, the level of mitochondrial membrane potential was rescued from loss under Se pretreatment as indicated by the decreased JC-1 monomer ratio (Figure 1(l) and 1(m)). In addition, ATP production assay showed that Se markedly boosted ATP generation in TBHP-challenged NPCs (Figure 1(n)).

### 3.3. Se Ameliorated TBHP-Induced Apoptosis and ECM Degeneration of NPCs

**Figure 2:**
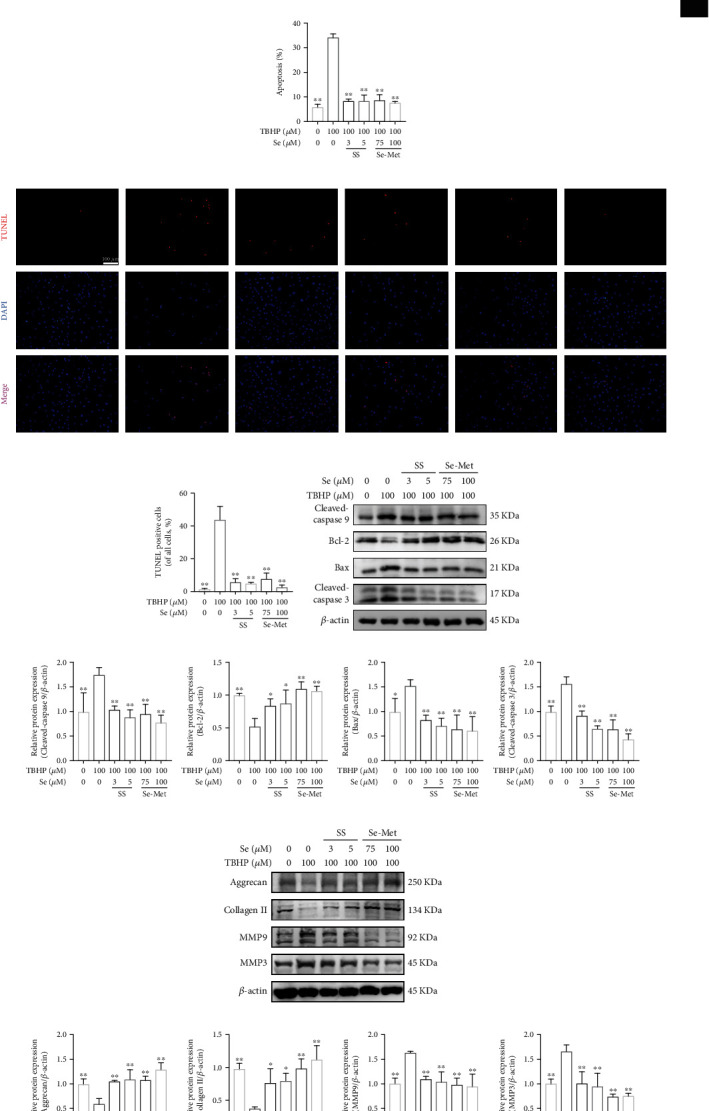
Se ameliorated TBHP-induced apoptosis and ECM degeneration of NPCs. (a, b) Apoptosis of the NPCs detected by flow cytometry analysis. (c, d) Typical fluorescence photomicrograph and quantitation of TUNEL staining of NPCs (scale bar: 100 *μ*m). (e, f) Representative western blot bands and quantitation of the expression of cleaved-caspase 9, cleaved-caspase 3, Bax, and Bcl-2 in the NPCs. (g, h) Representative western blot bands and quantitation of the expression of anabolic mediators (aggrecan and collagen II) and catabolic mediators (MMP3 and MMP9). The data are represented as the mean ± SD from at least 3 independent experiments. ^∗^*P* < 0.05 and ^∗∗^*P* < 0.01 vs. the TBHP group.

After treatment with TBHP (100 *μ*M) for 6 h, the apoptosis rate of NPCs rose to over 30% (Figures 2(a) and 2(b)), which was significantly higher than the control group (*P* < 0.01). However, pretreatment with Se inhibited the increase of apoptosis rate obviously, as indicated by flow cytometry analysis (Figures 2(a) and 2(b)). Consistently, TUNEL staining demonstrated that Se attenuated TBHP-induced NPCs apoptosis (Figures 2(c) and 2(d)).

As shown in Figures 2(e) and 2(f), TBHP treatment resulted in the upregulation of cleaved-caspase 9, cleaved-caspase 3, and Bax and the downregulation of Bcl-2, whereas Se treatment reversed these changes. The balance of ECM metabolism was also evaluated, and the results indicated that the expression of anabolic mediators (aggrecan and collagen II) decreased and catabolic mediators (MMP3, MMP9) augmented after TBHP treatment, while Se remarkably inverted these variations (Figures 2(g) and 2(h)).

### 3.4. Se Suppressed Mitochondrial Fission in TBHP-Exposed NPCs

**Figure 3:**
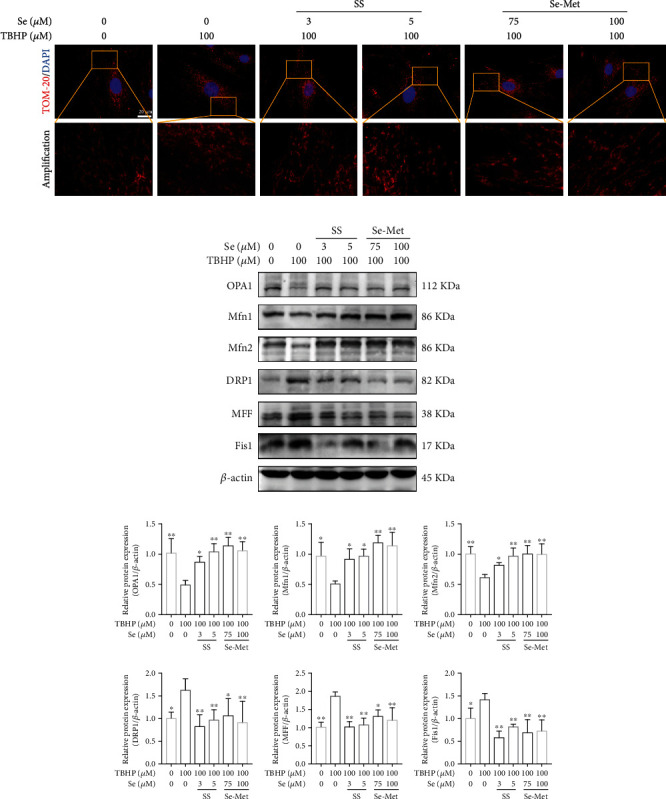
Se suppressed mitochondrial fission in TBHP-Exposed NPCs. (a) Changes in mitochondrial morphology of NPCs observed by IF staining of TOM-20 and represented by fluorescence photomicrograph (scale bar: 20 *μ*m). (b, c) Representative western blot bands and quantitation of the expression of OPA1, Mfn1, Mfn2, DRP1, MFF, and Fis1. The data are represented as the mean ± SD from at least 3 independent experiments. ^∗^*P* < 0.05 and ^∗∗^*P* < 0.01 vs. the TBHP group.

The equilibrium between mitochondrial fusion and fission is crucially involved in cell survival. IF staining of TOM-20 showed that TBHP treatment resulted in an increased level of mitochondrial fission, as indicated by shortened mitochondrial length compared to the control group. Nevertheless, the change of mitochondria was significantly ameliorated in cells pretreated with Se (Figure 3(a)).

In the TBHP group, the expression levels of proteins responsible for mitochondrial fission (DRP1, MFF, and Fis1) were upregulated, while the expression levels of OPA1, Mfn1, and Mfn2 were remarkably reduced compared to the control group. However, Se treatment relieved TBHP-induced alterations of the above proteins (Figures 3(b) and 3(c)). Collectively, these results indicated that Se rectified the imbalance between mitochondrial fusion and fission caused by TBHP.

### 3.5. Se Exerted Protective Effects in TBHP-Exposed NPCs through Suppressing Mitochondrial Fission

**Figure 4:**
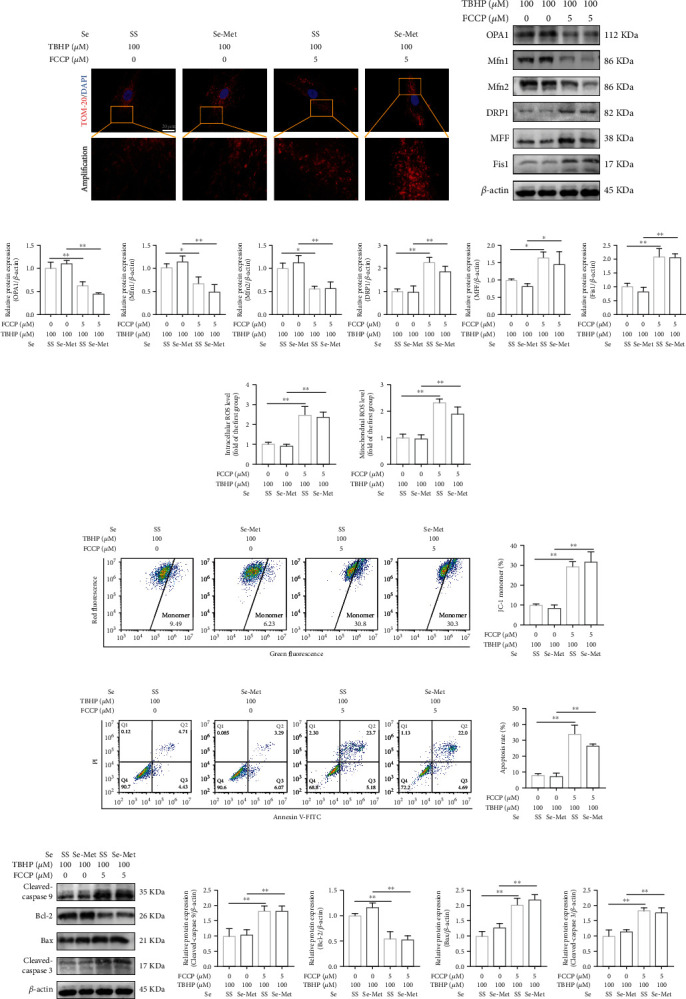
Se exerted protective effects in TBHP-exposed NPCs through suppressing mitochondrial fission. (a) Fluorescence photomicrograph of mitochondria of NPCs treated with FCCP and Se (scale bar: 20 *μ*m). (b, c) Representative western blot bands and quantitation of the expression levels of OPA1, Mfn1, Mfn2, DRP1, MFF, and Fis1 in NPCs treated with FCCP and Se. (d) The intracellular ROS levels in NPCs. (e) The mitochondrial ROS levels in NPCs. (f, g) Mitochondrial membrane potential levels measured by flow cytometry analysis. (h, i) Apoptosis of the NPCs detected by flow cytometry analysis. (j, k) Representative western blot bands and quantitation of the expression of apoptosis-related proteins. The data are represented as the mean ± SD from at least 3 independent experiments. Significant differences between groups are indicated as ^∗∗^*P* < 0.01, ^∗^*P* < 0.05.

Under the treatment of FCCP, Se failed to suppress excessive mitochondrial fission induced by TBHP, indicated by IF staining of TOM-20 (Figure 4(a)) and western blot (Figures 4(b) and 4(c)). Besides, FCCP partly blunted the beneficial effects of Se on alleviating the production of intracellular ROS (Figure 4(d) and Figure S1(c)) and mitochondrial ROS (Figure 4(e) and Figure S1(d)) and improving mitochondrial membrane potential levels (Figures 4(f) and 4(g)) in TBHP-exposed NPCs.

Through blocking the protective effects of Se, FCCP facilitated apoptosis of NPCs regardless of Se intervention, as proved by flow cytometry analysis (Figures 4(h) and 4(i)) and TUNEL staining (Figure S2(a)). Moreover, FCCP dramatically upregulated the expression levels of cleaved-caspase 9, cleaved-caspase 3, and Bax and downregulated Bcl-2 compared to the Se group (Figures 4(j) and 4(k)).

Taken together, these results revealed that Se alleviated oxidative stress, maintained the balance of mitochondrial homeostasis, and promoted cell survival against TBHP via suppressing mitochondrial fission.

### 3.6. Se Activated the Nrf2 Signaling Pathway in TBHP-Exposed NPCs

**Figure 5:**
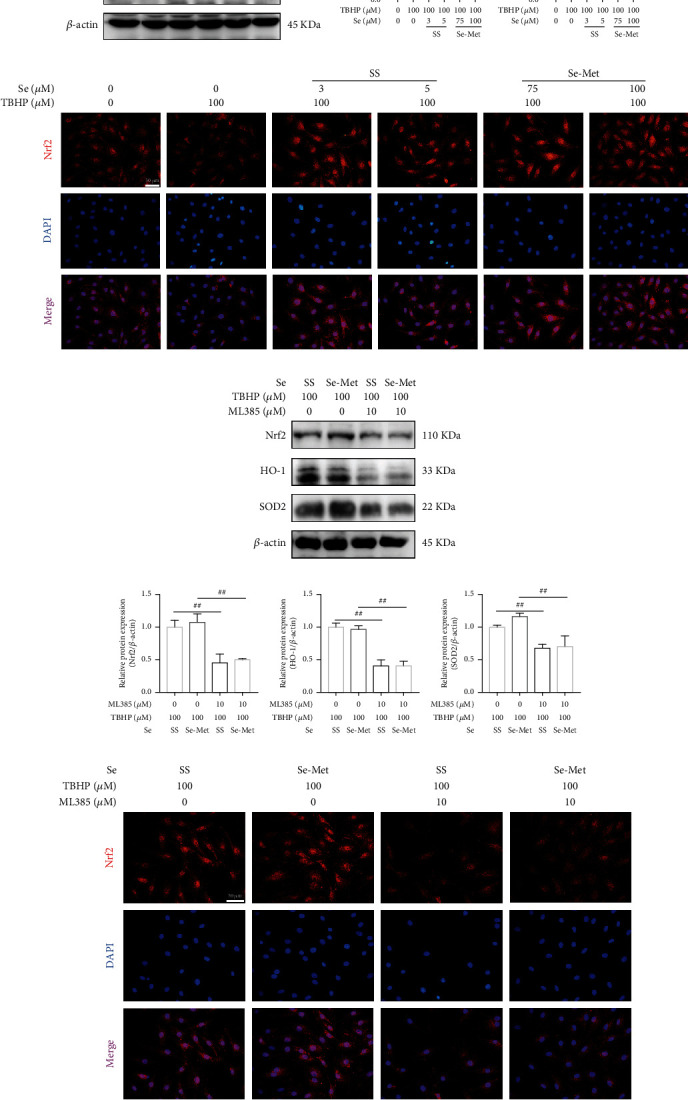
Se activated the Nrf2 signaling pathway in TBHP-exposed NPCs. (a, b) Representative western blot bands and quantitation of the expression of Nrf2 and HO-1 in NPCs. (c) Nucleus translocation and expression level of Nrf2 in NPCs (scale bar: 50 *μ*m). (d, e) Representative western blot bands and quantitation of the expression levels of Nrf2, HO-1, and SOD2 in NPCs treated with ML385 and Se. (f) Nuclear translocation and expression level of Nrf2 in NPCs treated with or without ML385 (scale bar: 50 *μ*m). The data are represented as the mean ± SD from at least 3 independent experiments. ^∗^*P* < 0.05 and ^∗∗^*P* < 0.01 vs. the TBHP group, ^#^*P* < 0.05 and ^##^*P* < 0.01.

We further explored the involvement of the Nrf2 antioxidative signaling pathway in the protective effects of Se on NPCs. TBHP downregulated the protein expression of Nrf2 and its downstream target HO-1 compared with the control group, while Se increased the expression of them in NPCs significantly (Figures 5(a) and 5(b)). Subsequently, IF staining of Nrf2 indicated that Se treatment distinctly facilitated the translocation of Nrf2 into the nucleus, as well as the expression of Nrf2 compared with the TBHP group (Figure 5(c)).

ML385 was employed to further confirm the effect of Se on activating the Nrf2 signaling pathway. ML385 greatly decreased the expression levels of Nrf2 and its downstream targets (HO-1 and SOD2) in comparison with the Se group (Figures 5(d) and 5(e)). Consistently, IF staining of Nrf2 showed that ML385 dramatically reduced the nucleus translocation of Nrf2, as well as the expression level of Nrf2 compared with the Se group (Figure 5(f)).

### 3.7. Se Suppressed Mitochondrial Fission and Cell Apoptosis through Activating the Nrf2 Pathway

**Figure 6:**
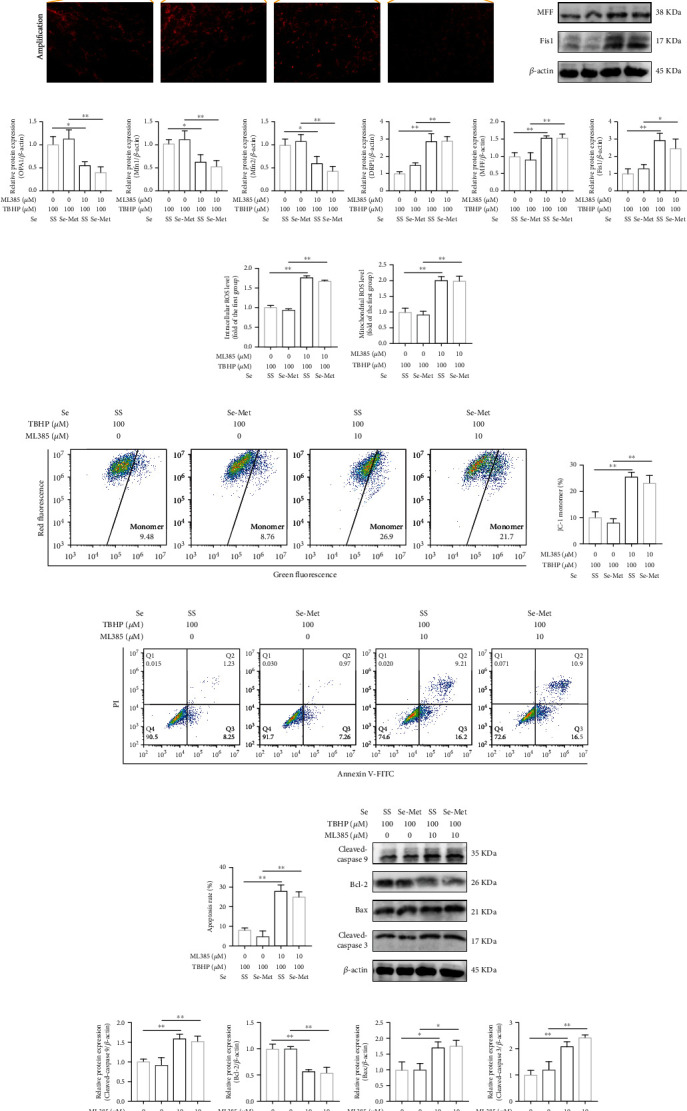
Se suppressed mitochondrial fission and cell apoptosis through activating the Nrf2 pathway. (a) Changes in mitochondrial morphology measured by fluorescence photomicrograph after treatment with ML385 and Se (scale bar: 20 *μ*m). (b, c) Representative western blot bands and quantitation of the expression levels of OPA1, Mfn1, Mfn2, DRP1, MFF, and Fis1 in NPCs. (d) The production of intracellular ROS in NPCs. (e) The production of mitochondrial ROS in NPCs. (f, g) Mitochondrial membrane potential measured by flow cytometry analysis. (h, i) Apoptosis of the NPCs detected by flow cytometry analysis. (j, k) Representative western blot bands and quantitation of the expression levels of cleaved-caspase 9, cleaved-caspase 3, Bax, and Bcl-2 in NPCs treated with or without ML385. The data are represented as the mean ± SD from at least 3 independent experiments. Significant differences between groups are indicated as ^∗∗^*P* < 0.01, ^∗^*P* < 0.05.

Under the treatment of ML385, Se failed to rectify the imbalance of mitochondrial dynamics induced by TBHP (Figure 6(a)). Consistently, ML385 promoted the expression levels of fission-related proteins (DRP1, MFF, and Fis1), while repressed OPA1, Mfn1, and Mfn2 compared to the Se group (Figures 6(b) and 6(c)). Flow cytometry analysis further revealed that ML385 partly abolished the effects of Se on reducing the production of intracellular ROS (Figure 6(d) and Figure S1(e)) and mitochondrial ROS (Figure 6(e) and Figure S1(f)) and maintaining mitochondrial membrane potential levels (Figures 6(f) and 6(g)) in TBHP-exposed NPCs.

Through impairing the protective effects of Se, ML385 triggered the apoptosis of NPCs as proved by flow cytometry analysis (Figures 6(h) and 6(i)) and TUNEL staining (Figure S2(b)). Additionally, ML385 partly abolished the antiapoptosis effect of Se on regulating the expressions of mitochondrial apoptosis-related proteins (cleaved-caspase 9, cleaved-caspase 3, Bax, and Bcl-2) (Figures 6(j) and 6(k)). Taken together, the Nrf2 pathway was involved in the cytoprotective effects of Se via regulating mitochondrial dynamics.

### 3.8. Se Ameliorated Puncture-Induced Rat IDD In Vivo

**Figure 7:**
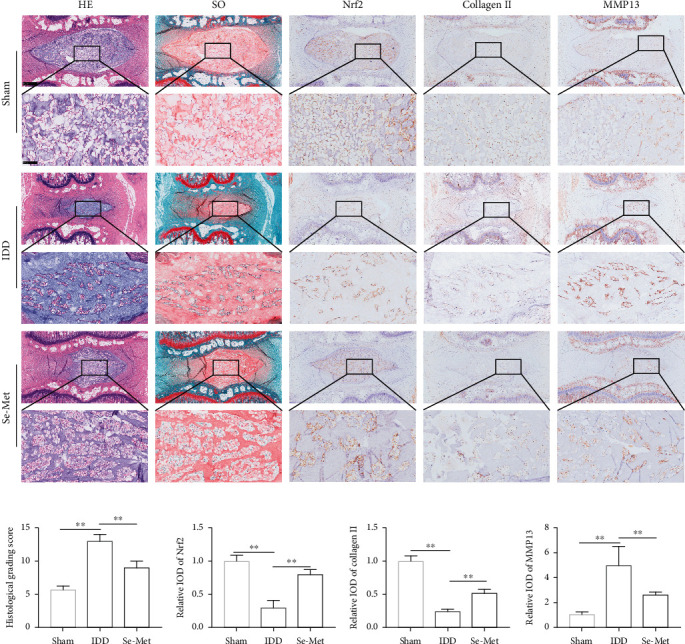
Se ameliorated puncture-induced rat IDD in vivo. (a) Histological analysis of the IVD samples (*n* = 7/group) by HE staining and SO staining, and the IHC staining of Nrf2, collagen II, and MMP13. (b) Histological grading scores assessed according to HE staining and SO staining. (c) IHC staining analysis for Nrf2, collagen II, and MMP13 expressions in the rat NP samples. Significant differences between groups are indicated as ^∗∗^*P* < 0.01, ^∗^*P* < 0.05.

To further verify the therapeutic effects of Se on IDD in vivo, we adopted a puncture-induced rat IDD model and administered Se-Met (2 mg/kg/day) intragastrically. As shown in Figure 7(a), HE and SO staining showed that obvious degenerative variations, including reduced NP size, collapsed disc height, significant loss of cells, and dense ECM, were observed in the IDD group compared to the sham group. Via assessing histological grading scores, we further proved that Se ameliorated puncture-induced IDD (Figure 7(b)). IHC staining showed that compared to the sham group, the levels of Nrf2 and collagen II decreased while the expression level of MMP13 increased in the IDD group. However, these changes were all weakened to a certain extent in the Se-Met group (Figures 7(a) and 7(c)).

## 4. Discussion

IDD is emerging as one of the most vital causes of LBP, which represents a major global health problem and leads to tremendous economic burdens. IVD is prone to degeneration due to the combination of environmental and genetic risk factors. Since NP serves as an important component of IVD, apoptosis and dysfunction of NPCs play crucial roles in the process of IDD. Despite extensive research, there remains an urgent need for exploring effective and specific therapeutic strategies to prevent or reverse the progression of IDD, owing to the incomplete elucidation of the underlying mechanisms. Hence, in the present study, we illustrated that Se ameliorated TBHP-induced oxidative stress and rectified the imbalance of mitochondrial dynamics, thereby protecting NPCs from mitochondrial apoptosis. Moreover, by the employment of ML385, we then clarified that the cytoprotective effects of Se on NPCs were related to the Nrf2 pathway. Consistent with in vitro experiments, the protective effects of Se-Met were confirmed in the puncture-induced rat IDD model.

Se is an indispensable metalloid trace element that is essential for cell survival. Given the important role in maintaining the optimal redox environment in cells and tissues, a growing body of evidence has implicated that Se is involved in various physiological functions, such as antioxidation, immunity, metabolism, and reproduction, exhibiting beneficial antiapoptosis effects [31–33]. Sun et al. employed SS to treat hydrogen peroxide-exposed rat cardiomyoblast cells and found that SS supplementation alleviated hydrogen peroxide-induced oxidative stress [40]. In this study, both SS and Se-Met were proved to alleviate TBHP-induced overproduction of intracellular ROS and elevated MDA levels, as well as upregulate the expression level of SOD2. Moreover, existing research revealed that Se could defend LLC-PK (1) cells against cadmium-induced apoptosis [41]. Our results indicated that TBHP-induced excessive apoptosis of NPCs could be reversed by Se treatment. In addition to Se content, the speciation of Se is also important. Recently, in comparison to traditional inorganic Se (SS), organic Se (Se-Met) has attracted increasing attention on account of its superior properties, including higher bioavailability, more rapid and efficient incorporation, and greater antioxidative capacity [42–44]. In line with these findings, both SS and Se-Met exerted protective effects on NPCs in our study, but Se-Met showed a wider biosafety range and lower cytotoxicity. Hence, only Se-Met was then further used in vivo to verify its amelioration of disc degeneration. The HE and SO staining showed that Se-Met significantly attenuated puncture-induced disc degeneration.

It is well established that a healthy pool of mitochondria, with a dynamic equilibrium between consistent fission and fusion, is an important guarantee for cellular survival by supplying adequate energy. When encountering adverse stimuli, mitochondrial dynamics was disrupted and resulted in excessive mitochondrial fission [27]. In this study, TBHP treatment resulted in an increased level of mitochondrial fission. Excessive mitochondrial fission is closely related to mitochondrial dysfunction. Li et al. indicated that dynamin-like protein 1-dependent mitochondrial fragmentation contributed to mitochondrial dysfunction and then resulted in neuron apoptosis [45]. After hypoxia, mitochondrial DRP1 was found to contribute to the immoderate opening of the mitochondrial permeability transition pore, ultimately resulting in mitochondrial dysfunction [46]. Here, the addition of FCCP showed that imbalanced mitochondrial dynamics contributed to the overproduction of mitochondrial ROS and decreased mitochondrial membrane potential levels. Kang et al. found that compression resulted in excessive mitochondrial fission, which triggers the overproduction of cellular and mitochondrial ROS, mitochondrial dysfunction, and apoptosis in human NP cells [27]. Our results showed that FCCP induced excessive mitochondrial fission, thereby contributing to apoptosis of NPCs regardless of the cytoprotective effects of Se. Moreover, suppressing mitochondrial fission and promoting mitochondrial fusion were proven to be beneficial to the survival of IVD cells [27, 47]. Hence, agents targeting mitochondrial fission are promising strategies in the treatment of IDD [20, 48].

Se was proved to provide antioxidative effects via activating the Nrf2 signaling pathway. A literature review reported that Se conferred protective effects on cardiovascular disease via several signaling pathways, among which the Nrf2 pathway plays a pivotal role in ameliorating oxidative stress [31]. In the in vitro study, Se notably upregulated the expression levels of Nrf2 and its downstream targets compared to the TBHP group. However, the employment of ML385 partially hindered the antioxidative effects of Se. Under stressed conditions, Nrf2 dissociates from the Keap1-Nrf2 complex and then translocates into the nucleus, eventually promoting the expression of cytoprotective genes that facilitate cell survival [24]. Consistently, IF staining of Nrf2 showed that Se facilitated nucleus translocation of Nrf2 compared to the TBHP group, while ML385 inhibited this process. Notably, a large body of studies supported that activation of the Nrf2 pathway by various drugs protected IVD cells from oxidative stress-induced apoptosis in vitro and attenuated the progression of IDD in vivo [49, 50]. Here, Se was proved to protect NPCs from TBHP-induced apoptosis. When ML385 was used to block the Nrf2 pathway, Se failed to rescue NPCs from apoptosis, indicating that Se ameliorated TBHP-induced oxidative stress and apoptosis via activating the Nrf2 pathway. Besides, Tang et al. revealed that the expression level of Nrf2 decreased in human degenerative NP tissue samples and that Nrf2 knockout also aggravated IDD [51]. Accordingly, the in vivo study showed that the expression level of Nrf2 decreased in the IDD group compared with the sham group, while it significantly increased in the Se-Met group.

The Nrf2 pathway is tightly associated with mitochondrial dynamics [52, 53]. Several lines of evidence indicated that activation of the Nrf2 pathway suppressed mitochondrial fission and promoted mitochondrial fusion through the degradation of DRP1 [54–56]. Moreover, metformin was reported to ameliorate lead-induced mitochondrial fragmentation via activating the AMPK/Nrf2 pathway [57]. Immoderate mitochondrial fission leads to disturbance of mitochondrial homeostasis, thereby triggering cell death. Here, we confirmed that Se maintained the balance of mitochondrial dynamics via activating the Nrf2 pathway. However, blocking the Nrf2 signaling pathway by ML385 markedly enhanced mitochondrial fission and suppressed mitochondrial fusion compared to the Se group.

There are some limitations in the current study. Although NP can exchange substances with the blood circulation through the osmosis of annulus fibrosus and cartilage endplate, the distribution concentration of Se-Met in NP is not clear by intragastric administration. Moreover, X-ray and MRI examinations of rat tails were not performed in this study, so it is difficult to directly reflect the degree of IDD in this paper.

In conclusion, our findings demonstrated that Se, both SS and Se-Met, could attenuate TBHP-induced NPC apoptosis by regulating oxidative stress and suppressing mitochondrial fission via activating the Nrf2 signaling pathway. Furthermore, Se-Met could reverse the pathological changes of disc in the puncture-induced IDD rat model. By emphasizing the prominent role of mitochondrial dynamics in IDD, the current study proposed that mitochondrial dynamics maybe a novel therapeutic target for IDD. Overall, our study suggested that of Se as a therapeutic strategy for IDD is promising.

## Data Availability

All data are available upon request.

## Abbreviations

IVD: Intervertebral disc
IDD: IVD degeneration
LBP: Low back pain
NPCs: Nucleus pulposus cells
Se: Selenium
SS: Sodium selenite
Se-Met: Selenomethionine
TBHP: Tert-butyl hydroperoxide
Nrf2: Nuclear factor erythroid 2-related factor 2
ECM: Extracellular matrix
NP: Nucleus pulposus
ROS: Reactive oxygen species
Keap1: Kelch-like ECH-associated protein 1
HO-1: Heme oxygenase-1
SOD2: Superoxide dismutase 2
DRP1: Dynamin-related protein 1
GPX: Glutathione peroxidase
TRXR: Thioredoxin reductase
FCCP: Carbonyl cyanide 4-(trifluoromethoxy) phenylhydrazone
CCK-8: Cell Counting Kit-8
RIPA: Radioimmunoprecipitation assay
SDS-PAGE: Sodium dodecyl sulfate-polyacrylamide gel
PVDF: Polyvinylidene difluoride
MFF: Mitochondrial fission factor
Fis1: Mitochondrial fission 1
OPA1: Optic atrophy1
Mfn1: Mitofusin 1
Mfn2: Mitofusin 2
MMP3: Matrix metalloproteinase 3
MMP9: Matrix metalloproteinase 9
TBST: Tris-buffered saline containing tween 20
ECL: Electrochemiluminescence
Calcein-AM: Calcein acetoxymethyl ester
PI: Propidium iodide
PBS: Phosphate-buffered saline
TUNEL: Terminal deoxynucleotidyl transferase biotin-dUTP nick end labeling
DAPI: 4′,6-diamidino-2-phenylindole
IF: Immunofluorescence
MDA: Malondialdehyde
HE: Hematoxylin and eosin
SO: Safranin O-fast green
IOD: Integrated optical density
SD: Standard deviation
ANOVA: Analysis of variance
NS: No statistically significant difference.

## Supplementary Materials

Supplementary Materials Figure S1: flow cytometry analysis of the production of intracellular ROS and mitochondrial ROS. (a) Se attenuated TBHP-induced increase of intracellular ROS measured by flow cytometry analysis. (b) Se attenuated TBHP-induced increase of mitochondrial ROS measured by flow cytometry analysis. (c) The production of intracellular ROS in NPCs treated with or without FCCP. (d) The production of mitochondrial ROS in NPCs treated with or without FCCP. (e) The production of intracellular ROS in NPCs treated with or without ML385. (f) The production of mitochondrial ROS in NPCs treated with or without ML385. Figure S2: the effect of Se on inhibiting apoptosis of NPCs was blocked by FCCP and ML385. (a) Typical fluorescence photomicrograph of TUNEL staining of NPCs treated with FCCP (scale bar: 100 *μ*m). (b) Typical fluorescence photomicrograph of TUNEL staining of NPCs after treatment with ML385 (scale bar: 100 *μ*m).

## References

[1] Andersson G. B. (1999). Epidemiological features of chronic low-back pain. *Lancet*.

[2] Maetzel A., Li L. (2002). The economic burden of low back pain: a review of studies published between 1996 and 2001. *Best Practice & Research. Clinical Rheumatology*.

[3] Luoma K., Riihimäki H., Luukkonen R., Raininko R., Viikari-Juntura E., Lamminen A. (1976). Low back pain in relation to lumbar disc degeneration. *Spine*.

[4] Chou D., Samartzis D., Bellabarba C. (2011). Degenerative magnetic resonance imaging changes in patients with chronic low back pain: a systematic review. *Spine*.

[5] Chen J., Xuan J., Gu Y. T. (2017). Celastrol reduces IL-1*β* induced matrix catabolism, oxidative stress and inflammation in human nucleus pulposus cells and attenuates rat intervertebral disc degeneration in vivo. *Biomedicine & Pharmacotherapy*.

[6] Clouet J., Vinatier C., Merceron C. (2009). The intervertebral disc: from pathophysiology to tissue engineering. *Joint, Bone, Spine*.

[7] Buckwalter J. A. (1995). Aging and degeneration of the human intervertebral disc. *Spine*.

[8] Wang F., Cai F., Shi R., Wang X. H., Wu X. T. (2016). Aging and age related stresses: a senescence mechanism of intervertebral disc degeneration. *Osteoarthritis and Cartilage*.

[9] Ding F., Shao Z. W., Xiong L. M. (2013). Cell death in intervertebral disc degeneration. *Apoptosis*.

[10] Song D., Ge J., Wang Y. (2021). Tea polyphenol attenuates oxidative stress-induced degeneration of intervertebral discs by regulating the Keap1/Nrf2/ARE pathway. *Oxidative Medicine and Cellular Longevity*.

[11] Dimozi A., Mavrogonatou E., Sklirou A., Kletsas D. (2015). Oxidative stress inhibits the proliferation, induces premature senescence and promotes a catabolic phenotype in human nucleus pulposus intervertebral disc cells. *European Cells & Materials*.

[12] Lu Y., Zhou L., He S., Ren H. L., Zhou N., Hu Z. M. (2020). Lycopene alleviates disc degeneration under oxidative stress through the Nrf2 signaling pathway. *Molecular and Cellular Probes*.

[13] Navarro-Yepes J., Burns M., Anandhan A. (2014). Oxidative stress, redox signaling, and autophagy: cell death versus survival. *Antioxidants & Redox Signaling*.

[14] Krupkova O., Handa J., Hlavna M. (2016). The natural polyphenol epigallocatechin gallate protects intervertebral disc cells from oxidative stress. *Oxidative Medicine and Cellular Longevity*.

[15] Feng C., Yang M., Lan M. (2017). ROS: crucial intermediators in the pathogenesis of intervertebral disc degeneration. *Oxidative Medicine and Cellular Longevity*.

[16] Ding F., Shao Z. W., Yang S. H., Wu Q., Gao F., Xiong L. M. (2012). Role of mitochondrial pathway in compression-induced apoptosis of nucleus pulposus cells. *Apoptosis*.

[17] Nasto L. A., Robinson A. R., Ngo K. (2013). Mitochondrial-derived reactive oxygen species (ROS) play a causal role in aging-related intervertebral disc degeneration. *Journal of Orthopaedic Research*.

[18] Westermann B. (2010). Mitochondrial fusion and fission in cell life and death. *Nature Reviews. Molecular Cell Biology*.

[19] Youle R. J., van der Bliek A. M. (2012). Mitochondrial fission, fusion, and stress. *Science*.

[20] Xu X., Wang D., Zheng C. (2019). Progerin accumulation in nucleus pulposus cells impairs mitochondrial function and induces intervertebral disc degeneration and therapeutic effects of sulforaphane. *Theranostics*.

[21] Wang Y., Zuo R., Wang Z. (2019). Kinsenoside ameliorates intervertebral disc degeneration through the activation of AKT-ERK1/2-Nrf2 signaling pathway. *Aging*.

[22] Motohashi H., Yamamoto M. (2004). Nrf2-Keap1 defines a physiologically important stress response mechanism. *Trends in Molecular Medicine*.

[23] Zhang M., Zhang B. H., Chen L., An W. (2002). Overexpression of heme oxygenase-1 protects smooth muscle cells against oxidative injury and inhibits cell proliferation. *Cell Research*.

[24] Kensler T. W., Wakabayashi N., Biswal S. (2007). Cell survival responses to environmental stresses via the Keap1-Nrf2-ARE pathway. *Annual Review of Pharmacology and Toxicology*.

[25] Dinkova-Kostova A. T., Abramov A. Y. (2015). The emerging role of Nrf2 in mitochondrial function. *Free Radical Biology & Medicine*.

[26] Yang Y., Luo L., Cai X. (2018). Nrf2 inhibits oxaliplatin-induced peripheral neuropathy via protection of mitochondrial function. *Free Radical Biology & Medicine*.

[27] Kang L., Liu S., Li J., Tian Y., Xue Y., Liu X. (2020). The mitochondria-targeted anti-oxidant MitoQ protects against intervertebral disc degeneration by ameliorating mitochondrial dysfunction and redox imbalance. *Cell Proliferation*.

[28] Wang K., Hu S., Wang B., Wang J., Wang X., Xu C. (2019). Genistein protects intervertebral discs from degeneration via Nrf2-mediated antioxidant defense system: an in vitro and in vivo study. *Journal of Cellular Physiology*.

[29] Rusetskaya N. Y., Fedotov I. V., Koftina V. A., Borodulin V. B. (2019). Selenium compounds in redox regulation of inflammation and apoptosis. *Biomeditsinskaya Khimiya*.

[30] Liu K., Ding T., Fang L. (2020). Organic selenium ameliorates Staphylococcus aureus-induced mastitis in rats by inhibiting the activation of NF-*κ*B and MAPK signaling pathways. *Frontiers in Veterinary Science*.

[31] Shalihat A., Hasanah A. N., Lesmana R., Budiman A., Gozali D. (2021). The role of selenium in cell survival and its correlation with protective effects against cardiovascular disease: a literature review. *Biomedicine & Pharmacotherapy*.

[32] Sakamoto M., Yasutake A., Kakita A. (2013). Selenomethionine protects against neuronal degeneration by methylmercury in the developing rat cerebrum. *Environmental Science & Technology*.

[33] Rayman M. P., Winther K. H., Pastor-Barriuso R. (2018). Effect of long-term selenium supplementation on mortality: results from a multiple-dose, randomised controlled trial. *Free Radical Biology & Medicine*.

[34] Zhang Y., Hu B., Wang M. (2020). Selenium protects against zearalenone-induced oxidative stress and apoptosis in the mouse kidney by inhibiting endoplasmic reticulum stress. *Oxidative Medicine and Cellular Longevity*.

[35] Zhang C., Lin J., Ge J. (2017). Selenium triggers Nrf2-mediated protection against cadmium-induced chicken hepatocyte autophagy and apoptosis. *Toxicology In Vitro*.

[36] Chen S., Lv X., Hu B. (2018). Critical contribution of RIPK1 mediated mitochondrial dysfunction and oxidative stress to compression-induced rat nucleus pulposus cells necroptosis and apoptosis. *Apoptosis*.

[37] Lu S., Song Y., Luo R. (2021). Ferroportin-dependent iron homeostasis protects against oxidative stress- induced nucleus pulposus cell ferroptosis and ameliorates intervertebral disc degeneration in vivo. *Oxidative Medicine and Cellular Longevity*.

[38] Chen D., Xia D., Pan Z. (2016). Metformin protects against apoptosis and senescence in nucleus pulposus cells and ameliorates disc degeneration in vivo. *Cell Death & Disease*.

[39] Han B., Zhu K., Li F. C. (2008). A simple disc degeneration model induced by percutaneous needle puncture in the rat tail. *Spine*.

[40] Sun W., Zhu J., Li S., Tang C., Zhao Q., Zhang J. (2020). Selenium supplementation protects against oxidative stress-induced cardiomyocyte cell cycle arrest through activation of PI3K/AKT. *Metallomics*.

[41] Zhou Y. J., Zhang S. P., Liu C. W., Cai Y. Q. (2009). The protection of selenium on ROS mediated-apoptosis by mitochondria dysfunction in cadmium-induced LLC-PK_1_ cells. *Toxicology In Vitro*.

[42] Takahashi K., Suzuki N., Ogra Y. (2017). Bioavailability comparison of nine bioselenocompounds in vitro and in vivo. *International Journal of Molecular Sciences*.

[43] Anan Y., Ohbo A., Tani Y., Ogra Y. (2014). Metabolic pathway of inorganic and organic selenocompounds labeled with stable isotope in Japanese quail. *Analytical and Bioanalytical Chemistry*.

[44] Suryo Rahmanto A., Davies M. J. (2011). Catalytic activity of selenomethionine in removing amino acid, peptide, and protein hydroperoxides. *Free Radical Biology & Medicine*.

[45] Li C., Wang D., Wu W. (2018). DLP1-dependent mitochondrial fragmentation and redistribution mediate prion- associated mitochondrial dysfunction and neuronal death. *Aging Cell*.

[46] Duan C., Kuang L., Hong C. (2021). Mitochondrial Drp1 recognizes and induces excessive mPTP opening after hypoxia through BAX-PiC and LRRK2-HK2. *Cell Death & Disease*.

[47] Wu W., Jing D., Huang X., Yang W., Shao Z. (2021). Drp1-mediated mitochondrial fission is involved in oxidized low-density lipoprotein-induced AF cella poptosis. *Journal of Orthopaedic Research*.

[48] Song Y., Lu S., Geng W. (2021). Mitochondrial quality control in intervertebral disc degeneration. *Experimental & Molecular Medicine*.

[49] Kang L., Liu S., Li J., Tian Y., Xue Y., Liu X. (2020). Parkin and Nrf2 prevent oxidative stress-induced apoptosis in intervertebral endplate chondrocytes via inducing mitophagy and anti-oxidant defenses. *Life Sciences*.

[50] Hua W., Li S., Luo R. (2020). Icariin protects human nucleus pulposus cells from hydrogen peroxide-induced mitochondria-mediated apoptosis by activating nuclear factor erythroid 2-related factor 2. *Biochimica et Biophysica Acta-Molecular Basis of Disease*.

[51] Tang Z., Hu B., Zang F., Wang J., Zhang X., Chen H. (2019). Nrf2 drives oxidative stress-induced autophagy in nucleus pulposus cells via a Keap1/Nrf2/p62 feedback loop to protect intervertebral disc from degeneration. *Cell Death & Disease*.

[52] Kang T. C. (2020). Nuclear factor-erythroid 2-related factor 2 (Nrf2) and mitochondrial dynamics/mitophagy in neurological diseases. *Antioxidants*.

[53] Riis S., Murray J. B., O'Connor R. (2020). IGF-1 signalling regulates mitochondria dynamics and turnover through a conserved GSK-3*β*-Nrf2-BNIP3 pathway. *Cell*.

[54] Zhu Y., Li M., Lu Y., Li J., Ke Y., Yang J. (2019). Ilexgenin A inhibits mitochondrial fission and promote Drp1 degradation by Nrf2-induced PSMB5 in endothelial cells. *Drug Development Research*.

[55] Sabouny R., Fraunberger E., Geoffrion M. (2017). The Keap1-Nrf2 stress response pathway promotes mitochondrial hyperfusion through degradation of the mitochondrial fission protein Drp 1. *Antioxidants & Redox Signaling*.

[56] Hou L., Zhang J., Liu Y. (2021). MitoQ alleviates LPS-mediated acute lung injury through regulating Nrf2/Drp1 pathway. *Free Radical Biology & Medicine*.

[57] Yang L., Li X., Jiang A. (2020). Metformin alleviates lead-induced mitochondrial fragmentation via AMPK/Nrf2 activation in SH-SY5Y cells. *Redox Biology*.

