# Supplementary material for: Selenium Attenuates TBHP-Induced Apoptosis of Nucleus Pulposus Cells by Suppressing Mitochondrial Fission through Activating Nuclear Factor Erythroid 2-Related Factor 2
Source: Oxid Med Cell Longev. 2022 Apr 11;2022:7531788. doi: 10.1155/2022/7531788 (PMC9017574; doi:10.1155/2022/7531788)
Supplement: Supplementary Materials — Figure S1: flow cytometry analysis of the production of intracellular ROS and mitochondrial ROS. (a) Se attenuated TBHP-induced increase of intracellular ROS measured by flow cytometry analysis. (b) Se attenuated TBHP-induced increase of mitochondrial ROS measured by flow cytometry analysis. (c) The production of intracellular ROS in NPCs treated with or without FCCP. (d) The production of mitochondrial ROS in NPCs treated with or without FCCP. (e) The production of intracellular ROS in NPCs treated with or without ML385. (f) The production of mitochondrial ROS in NPCs treated with or without ML385. Figure S2: the effect of Se on inhibiting apoptosis of NPCs was blocked by FCCP and ML385. (a) Typical fluorescence photomicrograph of TUNEL staining of NPCs treated with FCCP (scale bar: 100 μm). (b) Typical fluorescence photomicrograph of TUNEL staining of NPCs after treatment with ML385 (scale bar: 100 μm). [file 7531788.f1.docx]

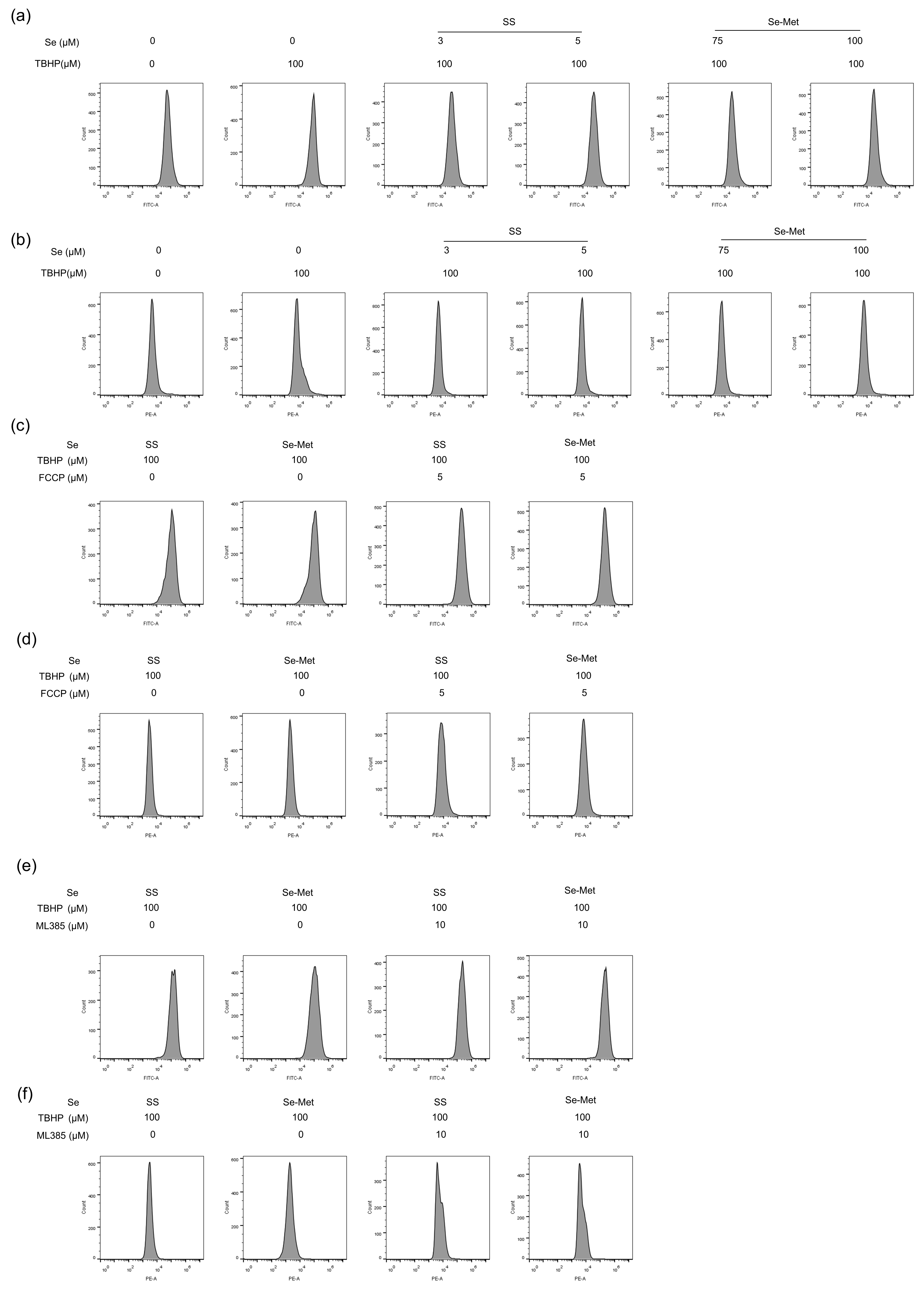


Figure S1 Flow cytometry analysis of the production of intracellular ROS and mitochondrial ROS. (a) Se attenuated TBHP-induced increase of intracellular ROS measured by flow cytometry analysis. (b) Se attenuated TBHP-induced increase of mitochondrial ROS measured by flow cytometry analysis. (c) The production of intracellular ROS in NPCs treated with or without FCCP. (d) The production of mitochondrial ROS in NPCs treated with or without FCCP. (e) The production of intracellular ROS in NPCs treated with or without ML385. (f) The production of mitochondrial ROS in NPCs treated with or without ML385.
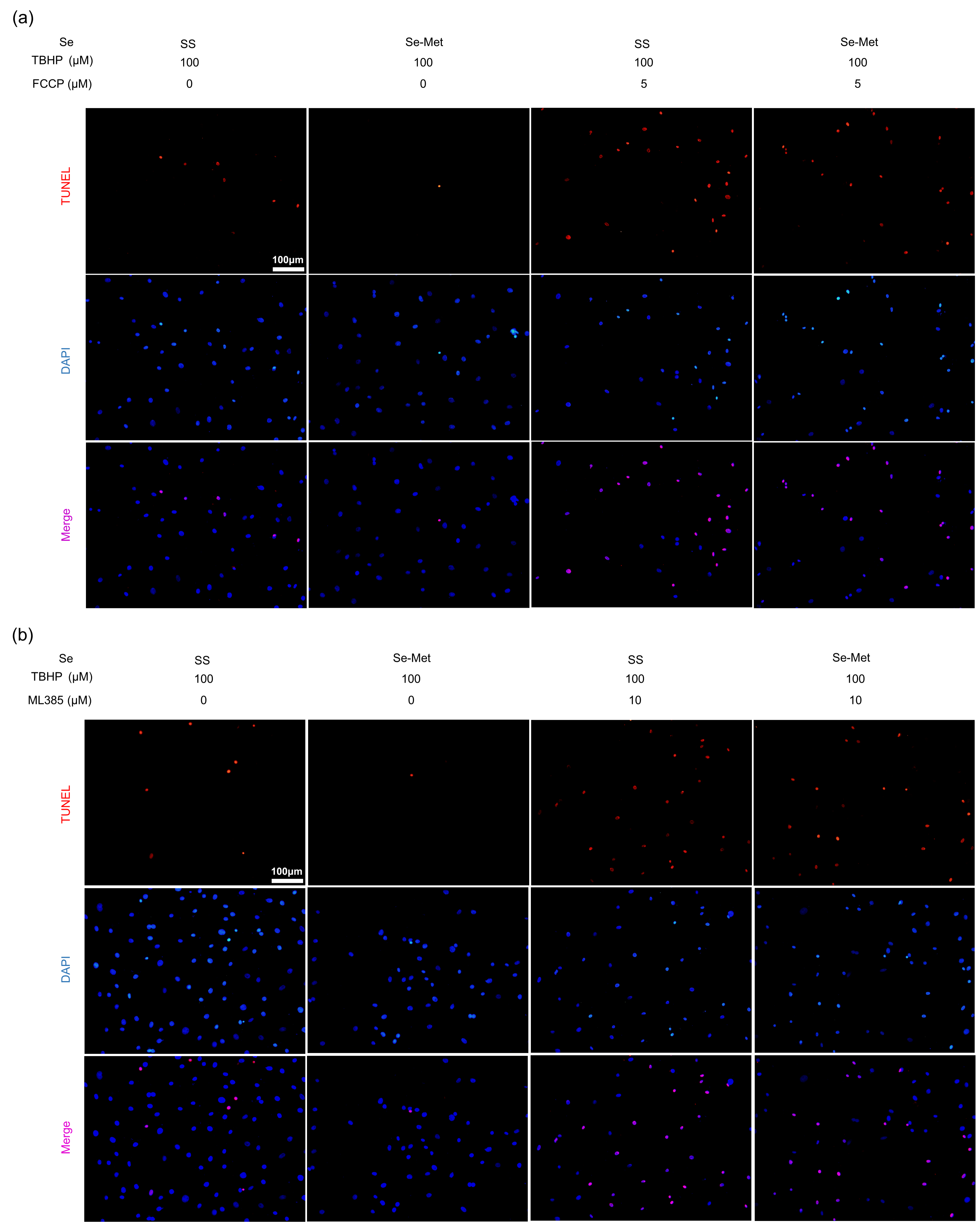


Figure S2 The effect of Se on inhibiting apoptosis of NPCs was blocked by FCCP and ML385. (a) Typical fluorescence photomicrograph of TUNEL staining of NPCs treated with FCCP (scale bar: 100 μm). (b) Typical fluorescence photomicrograph of TUNEL staining of NPCs after treatment with ML385 (scale bar: 100 μm).
